# Editorial: Advances in therapeutic cancer vaccines-mechanisms and development

**DOI:** 10.3389/fimmu.2026.1838269

**Published:** 2026-04-13

**Authors:** Mariam Mathew George, Cory A. Brennick, James J. Moon, Jayon Lihm, Hyejin Choi

**Affiliations:** 1 Sandra and Edward Meyer Cancer Center, Weill Cornell Medicine, New York, NY, United States; 2 Carole and Ray Neag Comprehensive Cancer Center, University of Connecticut School of Medicine, Farmington, CT, United States; 3 Department of Pharmaceutical Sciences, University of Michigan, Ann Arbor, MI, United States; 4 Memorial Sloan Kettering Cancer Center, New York, NY, United States; 5 Johnson & Johnson, Spring House, PA, United States

**Keywords:** bladder cancer, cancer, cancer vaccine platforms, lung cancer, pancreatic cancer, TCR-pMHC binding prediction, therapeutic cancer vaccine, triple negative breast cancer

Therapeutic cancer vaccines target established cancers primarily by expanding and diversifying tumor-specific T-cell responses against tumor-associated or tumor-specific antigens (1, 2). It has been increasingly recognized as a promising component of modern cancer immunotherapy given its abilities to induce durable tumor-reactive T cells associated with positive response and improved outcomes in clinical settings (3–7). The Research Topic *“Advances in Therapeutic Cancer Vaccines: Mechanisms and Development”* highlights recent progress in antigen discovery, vaccine platforms, and mechanistic insights that underlie the induction of more effective antitumor immune responses. Collectively, these articles highlight the importance of a deeper understanding of tumor–immune interactions for vaccine design, rational combinations with other therapeutic modalities, progress of diverse vaccine approaches, and clinical trials in various cancer types. Together, the contributions in this Research Topic illustrate a shift toward precision vaccine strategies tailored to individual tumor and immune contexts. This Research Topic reflects a field transitioning from experimental promise to translational reality in diverse cancer types.

Triple-negative breast cancer (TNBC) is known as an aggressive and heterogenous subtype prone to antigen-loss escape and inconsistent responses to immunotherapy (8–11). Lee et al. describe the development of “TNBCvax,” a multivalent peptide vaccine targeting TOP2A, HIF-1α, and IGF-1R for immunoprevention. The authors evaluate the vaccine in both syngeneic tumor graft and spontaneous genetically engineered TNBC murine models, demonstrating significant inhibition of tumor growth and delayed tumor onset compared with adjuvant controls. In several comparisons, the multivalent formulation outperformed single-antigen vaccines, supporting the rationale for broader antigen coverage. Mechanistically, TNBCvax induced robust Th1-skewed immune responses, as shown by increased IFN-γ production, enhanced CD8^+^ T cell infiltration, and elevated cytotoxic markers, such as granzyme B and TNF-α within tumors. The vaccine also promoted central memory T cell populations, suggesting potential durability of immune protection. Overall, this study provides preclinical evidence that multivalent vaccination can mitigate tumor heterogeneity and enhance antitumor immunity against TNBC. Future work will need to address translational considerations, including human antigen prevalence, HLA coverage, and integration with complementary immunotherapies.

A key challenge in vaccine development is the identification of optimal antigens, with in silico approaches increasingly used to evaluate and prioritize targets capable of eliciting robust T cell responses. Chao et al. addresses this by integrating advanced computational immunology with structural biology to enhance prediction of T cell receptor (TCR) interactions with peptide–major histocompatibility complex (pMHC) complexes. Precise TCR-pMHC recognition underpins effective T cell activation, which is essential for vaccine-mediated tumor eradication and long-term immunological memory. Building on breakthroughs in protein structure prediction, such as AlphaFold 3, the authors demonstrate how deep learning–driven modeling can distinguish immunogenic epitopes from non-functional sequences in silico, offering a scalable alternative to labor-intensive experimental methods. Their findings highlight both the promise and current limitations of artificial intelligence (AI) and machine learning (ML) tools in guiding antigen selection, TCR specificity prediction, and the design of high-affinity T cell-based therapies. By providing a computational framework to rationally prioritize vaccine targets and optimize T cell engagement, this perspective helps integrate mechanistic insights with the broader goals of personalized vaccine development and precision immunotherapy.

Despite the challenges of developing effective therapeutic vaccines, progress has been made across multiple cancer types. Li et al. reviews the recent advancements in bladder cancer immunotherapy, with a particular focus on optimizing Bacillus Calmette-Guérin (BCG) therapy and developing next-generation vaccine platforms. The authors dive into the clinical importance of intravesical BCG therapy for bladder cancer while highlighting its persistent limitations, including toxicity, treatment failure, and supply constraints. These shortcomings provide a strong rationale for innovation. The authors provide a systematic comparison of emerging vaccine strategies, spanning recombinant and nanoparticle-enhanced BCG, peptide-based vaccines, dendritic cell vaccines, viral vectors, and nucleic acid platforms. They integrate mechanistic insights with clinical trial data, assessing immunogenicity, feasibility, and translational readiness. Notably, the review emphasizes the growing role of personalized and combination approaches, particularly vaccines paired with immune checkpoint inhibitors, to overcome tumor heterogeneity and immune suppression. Overall, this article is an informative synthesis that effectively bridges historical BCG therapy with emerging immunotherapeutic strategies, making it a valuable resource for both researchers and clinicians in bladder cancer immunology.

Wang et al. provide an overview of therapeutic cancer vaccines for pancreatic ductal adenocarcinoma (PDAC), a malignancy with rising incidence, high mortality, and limited benefit from current systemic therapies. They outline the immunologic basis of vaccination and summarize clinical efforts with cell-based, nucleic acid–based, and peptide vaccines, including WT1- and MUC1-pulsed dendritic cells, whole–tumor-cell products such as GVAX and algenpantucel-L, and emerging mRNA and neoantigen-targeted platforms. Early-phase trials show that these strategies can elicit tumor-specific T cell responses and occasionally permit conversion surgery, yet survival gains remain modest due to barriers such as low mutational burden, a profoundly immunosuppressive microenvironment, manufacturing and technological hurdles, suboptimal preclinical models, and layered immunotherapy resistance. To overcome these limitations, the authors advocate combining vaccines with chemotherapy, checkpoint blockade, and stromal or myeloid targeting, while leveraging advances in sequencing, delivery systems, and nanotechnology to enable personalized vaccination strategies. Sun et al. complement this perspective by reviewing therapeutic vaccines for lung cancer, tracing progress from GVAX whole–tumor-cell vaccines to contemporary mRNA platforms. They classify approaches by antigen types and delivery modalities, encompassing tumor cell, autologous dendritic cell, mRNA, peptide/protein, and viral-vector vaccines directed against antigens such as tumor cell lysates, ALK, GD3, MAGE-1, and neoantigens. Spanning preclinical and predominantly early-phase clinical studies, this work highlights active development pipelines, advances in antigen screening, innovative delivery platforms, novel administration strategies, and rational combinations with checkpoint inhibitors, chemotherapy, targeted agents, and multi-target immunomodulators. Together, these articles offer a concise, forward-looking synthesis of therapeutic vaccine development in PDAC and lung cancer.

Together, the contributions in this Research Topic underscore how therapeutic cancer vaccines are moving beyond proof-of-concept toward increasingly sophisticated, mechanism-guided interventions that can be tailored to distinct tumor ecosystems and clinical settings. By spanning preclinical model development, computational antigen discovery, and disease-specific translational advances, these studies collectively chart a roadmap for integrating vaccination into multi-modal immunotherapy strategies. Continued progress will depend on strengthening the dialogue between basic, computational, and clinical research, but the work assembled here offers a compelling glimpse of how rationally designed cancer vaccines may soon become integral to personalized cancer care.
